# Reinvestigation of the Cd–Gd phase diagram

**DOI:** 10.1016/j.jallcom.2014.07.211

**Published:** 2014-12-25

**Authors:** Thomas L. Reichmann, Herbert Ipser

**Affiliations:** Department of Inorganic Chemistry (Materials Chemistry), University of Vienna, 1090 Wien, Austria

**Keywords:** Intermetallics, Phase transformation, Heat treatment, Microstructure, Diffraction (X-ray), Differential thermal analysis

## Abstract

The complete Cd–Gd equilibrium phase diagram was investigated by a combination of powder-XRD, SEM and DTA. All previously reported phases, i.e., CdGd, Cd_2_Gd, Cd_3_Gd, Cd_45_Gd_11_, Cd_58_Gd_13_, and Cd_6_Gd, could be confirmed. In addition, a new intermetallic compound with a stoichiometric composition corresponding to “Cd_8_Gd” was found to exist. It was obtained that “Cd_8_Gd” decomposes peritectically at 465 °C. Homogeneity ranges of all intermetallic compounds were determined at distinct temperatures. In addition, the maximum solubilities of Cd in the low- and high-temperature modifications of Gd were determined precisely as 4.6 and 22.6 at.%, respectively. All invariant reaction temperatures (with the exception of the formation of Cd_58_Gd_13_) as well as liquidus temperatures were determined, most probably, Cd_58_Gd_13_ is formed in a peritectoid reaction from Cd_45_Gd_11_ and Cd_6_Gd at a temperature below 700 °C.

## Highlights

• The complete Cd–Gd phase diagram was reinvestigated.
• A new compound “Cd_8_Gd” was found.
• Homogeneity ranges were determined properly.
• The melting behaviour of Cd_58_Gd_13_ was altered.

## 1 Introduction

For an efficient use of nuclear energy, reprocessing of nuclear waste should be balanced with an adequate waste management. Actually, low-level and intermediate-level radioactive waste is mostly stored in interim storage facilities or deposited in geological repositories. The situation concerning disposal of high-level nuclear waste is more serious. In fact, deposition of this waste in e.g., geological repositories does not solve the problem but defers it to further generations. Hence, disposal of nuclear waste turned more and more into a question of sustainability. In fact, no geological repository for high-level waste is presently available and thus it is actually stored on-site.

To lower the amount of radioactive waste, separation techniques are currently employed to selectively reprocess spent nuclear fuels. Reprocessing is mostly performed by means of solvent extraction of actinides using tributyl phosphate (TBP), known as hydro-metallurgical technique or aqueous reprocessing [1]. To evade some of the key problems of these aqueous methods, pyro-metallurgical techniques have been developed [2]. The central part of pyro-metallurgical reprocessing is the electro-refining step where a liquid Cd cathode is used to selectively extract minor actinides from the irradiated metallic fuel. The theoretical feasibility was described repeatedly in literature, see e.g., Refs. [3], [4], [5]. One of the key problems of this method is the clean separation of actinides from a salt molten solution which also contains a considerable amount of lanthanides. Moriyama et al. [6] determined that the separation factors, which are an indicator for extractability, are quite different between actinides and lanthanides and are predominantly dependent on the employed liquid metal which is preferentially Cd [7]. Therefore, a detailed knowledge of the respective Cd-RE (RE…rare earth element) phase diagrams as well as of the thermodynamic stabilities of the corresponding intermetallic compounds is of great importance. This was the reason for initiating a series of thermodynamic and phase diagram studies of different Cd-RE systems in our laboratory (*cf.* Refs. [8], [9], [10], [11], [12], [13]).

Concerning the system Cd–Gd, a complete version of the phase diagram was available from literature, given by Bruzzone et al. [14]. The authors presented phase equilibria and melting behaviour in the complete composition range and showed the occurrence of altogether six intermetallic compounds. They identified three new phases, i.e., Cd_3_Gd, Cd_4∼_Gd and Cd_4.5∼_Gd, of which the latter two were reported to be isomorphous with Pt_5_Zn_21_ [15] and Gd_13_Zn_58_ [16], respectively. In addition, they confirmed the existence of CdGd, Cd_2_Gd and Cd_6_Gd which had already been reported previously [25], [27], [29]. Moreover, a polymorphic transformation of Cd_2_Gd into a high temperature modification (β-Cd_2_Gd) at 995 °C was indicated by Bruzzone et al. [14], but no information on the corresponding crystal structure was given since quenching of this high temperature phase (β-Cd_2_Gd) was not possible. It was further stated that no detectable range of solid solubility was observed for any of the phases except for the high temperature allotropic modification of Gd. By addition of Cd, β-Gd could be stabilized down to 725 °C where it decomposes in a eutectoid reaction at 16 at.% Cd. It was shown that β-Gd forms a eutectic reaction with CdGd at 27 at.% Cd and 920 °C, a temperature where 22 at.% Cd are dissolved. The only congruently melting phase in this system is CdGd with a melting point of 1170 °C at the stoichiometric composition. All other compounds are formed incongruently: β-Cd_2_Gd (1010 °C), Cd_3_Gd (815 °C), Cd_4∼_Gd (i.e., Cd_45_Gd_11_, 806 °C), Cd_4.5∼_Gd (i.e., Cd_58_Gd_13_, 803 °C), and Cd_6_Gd (716 °C). Hence, the liquidus curve continuously decreases from 1170 to 316 °C, which corresponds to the eutectic formed between Cd and Cd_6_Gd at 97.5 at.% Cd.

The solubility of Gd in liquid Cd was extrapolated from DTA results but does not correspond to data presented earlier by Johnson [17] who had determined the solubility of Gd in liquid Cd by chemical analysis of filtered samples between 324 and 500 °C. From these results Johnson derived much lower solubility limits within this temperature range, i.e., 0.17–1.45 at.% Gd. In a subsequent study, Roshchina and Bayanov [18] employed an emf method to determine activities of Gd in liquid Cd. From their emf signals the solubility of Gd in liquid Cd was estimated between 390 and 528 °C. The values were given within 2.4 and 3.5 at.% Gd which agrees with the data of Bruzzone et al. [14].

Based on a combination of all available data, Gschneidner and Calderwood [19] presented an assessment of the Cd–Gd phase diagram which corresponds to the version of Bruzzone et al. [14]. Gschneidner and Calderwood described the phases Cd_4∼_Gd and Cd_4.5∼_Gd as Cd_45_Gd_11_ and Cd_58_Gd_13_, respectively, which is a consistent notation for these compounds also in other Cd-RE systems. Moreover, they raised the isothermal reaction temperature of the eutectic reaction L ⇄ CdGd + β-Gd to 937 °C which is 17 °C higher than the value given by Bruzzone et al. [14]. Obviously, the latter authors used for sample preparation Gd metal with a melting point exactly 17 °C lower than the accepted value from Gschneidner and Calderwood [20]. In a subsequent study, Tang and Gschneidner [21] performed DTA measurements of rapidly quenched as-cast alloys and determined the eutectoid transformation from low to high temperature Gd at 738 °C which is 13 °C higher than the value given by Bruzzone et al. [14]. Tang and Gschneidner even obtained lattice parameters of solid solutions of Cd in β-Gd. Through a linear extrapolation they determined the lattice parameter of pure β-Gd, given as 3.99 ± 0.04 Å. Based on the results of Refs. [14], [17], [22], Kurata and Sakamura [23] made a CALPHAD-type optimization between 0 and 60 at.% Cd. All intermetallic compounds were modelled as stoichiometric line compounds.

Since all intermetallic Cd–Gd compounds have isomorphous homologues in other Cd-RE systems as already mentioned by Gschneidner and Calderwood [24], their crystal structures are sufficiently well known. A list of crystallographic data concerning all binary compounds relevant for the current work is shown in Table 1 together with phase boundaries at 500 °C from Reichmann et al. [13] and corresponding references [14], [44]. No significant homogeneity ranges of these phases were given in literature up to now. Concerning the Cd–Gd phases, vapour pressure measurements have been carried out by Reichmann et al. [13] and phase boundaries were derived at 500 °C, see Table 1. Significant solubility limits were determined for at least five compounds whereas Cd_3_Gd was described as a line compound.

**Table 1:** Crystal structure data of binary compounds in the Cd–Gd system; phase boundaries at 500 °C are given according to Ref. [13].

Phase	Lattice parameter (Å)	Phase boundaries (at.% Cd)	Structure type	Space group	References
“Cd_8_Gd”	–	–	–	–	This work
Cd_6_Gd	*a *= 15.441	84.8–85.7	Cd_6_Y	Im3¯	[44]
Cd_58_Gd_13_	*a *= 15.40	81.3–81.7	Pu_13_Zn_58_	*P*6_3_/*mmc*	[14]
	*c *= 15.28				
Cd_45_Gd_11_	*a *= 21.603	79.5–80.5	Cd_45_Sm_11_	$F\overset{¯}{4}3m$	[14]
Cd_3_Gd	*a *= 6.621	74.9–75.1	Ni_3_Sn	*P*6_3_/*mmc*	[14]
	*c = *4.933				
α-Cd_2_Gd	*a *= 4.941	65.3–67.1	AlB_2_^a^	P6/mmm^a^	[14]
	*c *= 3.467				
β-Cd_2_Gd^b^	–	–	–	–	[14]
CdGd	*a *= 3.750	49.0–51.0	CsCl	$\mathrm{Pm}\overset{¯}{3}m$	[14]

a Actually described with space group $P\overset{¯}{3}m1$ (CeCd_2_-type) by Ref. [14] but modified according to the results of the present study.

b High temperature modification formed at 995 °C [14].

Compounds with the respective composition ratio 1:1 were found in most systems between Cd and RE elements, and the system Cd–Gd is no exception [26], [27], [28]. As all other homologues, CdGd is crystallizing in the CsCl structure (*B2*) which is an ordered variety of the *bcc* W-structure of β-Gd. As it was recently described in the Cd-Pr phase diagram, CdPr can be built up by substituting Pr sites in β-Pr with Cd atoms [10]. Actually, the two-phase field β-Pr + CdPr can be treated as a miscibility gap which breaks off a continuous solid solution between both phases, a situation which seems to be quite similar in the Cd–Gd phase diagram (see Section 3.3. below).

The next compound richer in Cd is Cd_2_Gd (i.e., α-Cd_2_Gd, structure type *C*6). Compounds with the general formula Cd_2_RE were investigated extensively. They crystallize either in space-group *P*6/*mmm* or $P\overset{¯}{3}m1$ [29], [30], [31], [32], [33]. The only exceptions are Cd_2_Eu and Cd_2_Yb for which space group *Imma* [34] and P6_3_/mmc [35] were found. Compounds with space-group symmetry *P*6/*mmm* adopt the simple AlB_2_ structure type (Pearson code *hP*3) with Wyckoff sequence *d a*; the site positions are (1/3 2/3 1/2) and (0 0 0). The compounds with space-group $P\overset{¯}{3}m1$ maintain the Pearson code and Wyckoff sequence, the site positions are (1/3 2/3 *z*) and (0 0 0). However, the *z* parameter of the site 2(*d*) varies either between 0.042 and 0.080 or between 0.42 and 0.43. Phases adopting *z* parameters around 0.42 can be considered as having a distorted atomic arrangement from that found in space group *P*6/*mmm*. Curiously, both atomic arrangements with symmetry $P\overset{¯}{3}m1$ are currently known as the Cd_2_Ce-type.

Iandelli and Palenzona [29] described Cd_2_Gd to crystallize in the Cd_2_Ce-type where Cd occupies the site 2(*d*) (1/3 2/3 *z*) with *z* = 0.42. The authors outlined that this atomic arrangement corresponds to a completely disordered mixture of the simple AlB_2_-type and the CdI_2_-type structures. From a single-crystal investigation of the Cd_2_Pr compound [10], it was obtained that this phase crystallizes in the simple AlB_2_-type with space group *P*6/*mmm* and the site positions (1/3 2/3 1/2) and (0 0 0). It was even found in the present study, that Cd_2_Gd (i.e., α-Cd_2_Gd) should crystallize in the simple AlB_2_-type, compare 3.1.

The compound Cd_3_Gd was first described by Bruzzone et al. [14] to crystallize in the Ni_3_Sn structure (*D*0_19_). Subsequent single-crystal measurements were performed by Bruzzone et al. [36] to determine crystal structure data in detail. Compounds with the composition ratio 3:1 were found in most of the phase diagrams of Cd with rare earth elements and appear with three different structure-types i.e., BiF_3_, Ni_3_Sn and ErCd_3_ [24]. With the exception of Cd_3_Tb, which either crystallizes in the hexagonal Ni_3_Sn or orthorhombic ErCd_3_ structure [36], Cd_3_Gd is the only compound preferring the hexagonal cell (P6_3_/mmc).

In the very narrow composition range 79–82 at.% Cd, two intermetallic compounds with rather big unit cells are occurring: Cd_45_Gd_11_ (*cF*448) and Cd_58_Gd_13_ (*hP*142) [14]. Cd_45_Gd_11_ is isostructural with Cd_45_Sm_11_, which was investigated by single-crystal X-ray diffraction [37]. This structure type can be described by the so-called cluster concept originally adopted by Bradley and Jones [38]. Cd_45_Gd_11_ is thus an arrangement of 16 clusters of two types which are distributed along a NaTl-type unit cell. The two types of clusters which build up the Cd_45_Gd_11_ cell are related to clusters found in γ-brass phases and in α-Mn, respectively.

Cd_58_Gd_13_ forms a complex atomic arrangement that is isotypic to Cd_58_RE_13_ where RE stands for: Y, La, Ce, Pr, Nd, Sm and Eu [33], [36], [39], [40]. Recently, Piao et al. [41] have synthesised also Cd_58_Dy_13_ and Cd_58_Tb_13_ but observed differences to the archetype structure. The basically hexagonal structure type contains various building blocks going along with extensive order–disorder phenomena resulting in distinct superstructures. Partly, an incommensurate behaviour is observed. More recently, Piao et al. [42] discussed for the compound Ce_12.60_Cd_58.68_ a metrically commensurate representative which obviously represents a lock-in phase.

The compound richest in Cd is Cd_6_Gd. Johnson et al. [25] presented cell parameters for a number of compounds with the composition ratio 6:1. No additional information was given except that all homologues seem to be isomorphous. Subsequently, Larson and Cromer [43] investigated the crystal structure of Cd_6_Y using single-crystal X-ray diffraction. They reported that the crystal structure is isotypic to Ru_3_Be_17_ but with an additional Cd position in a 24-fold position and the site occupation factor of 0.33. More recently, Gomez and Lidin [44] employed single-crystal X-ray diffraction to investigate disorder phenomena in MCd_6_ (M = Pr, Nd, Sm, Eu, Gd, Dy, Yb, Y and Ca). In the course of their study they could compare the different types of disorder of the central Cd_4_ tetrahedra, which belong to the additional Cd position introduced by Larson and Cromer. According to Gomez and Lidin, the respective Cd position is somehow split and manifests itself as smeared out electron density. Their corresponding structural data were taken for the present refinements.

Due to some discrepancies concerning the published phase diagram data, it was the aim of the present work to provide an experimental re-investigation of the Cd–Gd phase diagram. In addition, these data can serve as input into a CALPHAD-type optimization of the Cd–Gd system. This would contribute to a better understanding of the distribution behaviour of Gd in the electro-refining cell described above.

## 2 Experimental

All samples were prepared from pure elements, using cadmium shot (99.9999%, AlfaAesar, Johnson Matthey Chemicals, Karlsruhe, Germany) and gadolinium pieces (99.9%, Smart Elements, Vienna, Austria; 99.9%, Goodfellow, Cambridge, UK). The surface of the Gd pieces was filed to remove the corresponding oxide layer. The elements were weighed with a semi-micro balance to an accuracy of about ±0.5 mg which corresponds to an actual accuracy of ±0.01 at.%. Typically, samples with a total mass of 1 g were produced. To prevent Gd from oxidation, the whole sample preparation was carried out in a glove box, filled with Ar (oxygen and water level: <1 ppm each). The metals were placed in Ta crucibles which were designed in our laboratory and subsequently enclosed by means of arc welding under an Ar atmosphere of 0.28 bar.

For equilibration, the crucibles were sealed into silica glass tubes under dynamic vacuum of better than 10^−2^ mbar. Afterwards, sample preparation was done according to different heating procedures to achieve optimal homogenisation. Whereas the melting points of these elements differ noticeably, Cd has a rather high vapour pressure and a low boiling point, i.e., 767 °C [45]. This can cause a severe Cd pressure inside the crucibles, when heating the samples too fast to temperatures above 767 °C. Thus, all samples were slowly heated (0.5 K/min) to temperatures above the melting point of Cd and held for one day, which allowed a more or less complete reaction of Cd with Gd before reaching elevated temperatures. All heat treatments are listed in Table 2. It was observed that this pre-heating step had a decisive influence on the quality of the final samples: several times it was observed that samples with rather high Cd contents, heated too fast, showed a drastic expansion of the Ta crucibles. Sometimes, condensed Cd was found at the inner top of the Ta crucibles, resulting in a large deviation of the corresponding sample compositions. This behaviour could be avoided by a slow heating rate.

**Table 2:** Experimental phase compositions and lattice parameters of selected Cd–Gd samples.

Sample/nom. comp. (at.%)	Phase analysis			SEM (EDX)	
	Heat treatment *T* (°C); duration; *T*_max_ (°C)	Phase	Lattice parameter (Å)	Cd (at.%)	Gd (at.%)
1	300; 3 months; 800	Cd	*Too ductile*	100	0.0
Cd_98_Gd_2_		“Cd_8_Gd”		88.7	11.3
2	300; 2 months; 800	Cd	*a *= 2.9792(1), *c *= 5.6130(4)	100	0.0
Cd_90_Gd_10_		“Cd_8_Gd”	*No structure data available*	88.7	11.3
		Cd_6_Gd	*a *= 15.5263(4)	85.5	14.5
3	700; 3 months; 950	Cd_6_Gd	*a *= 15.5226(3)	85.2	14.8
Cd_83.5_Gd_16.5_		Cd_45_Gd_11_	*a = *21.6003(3)	81.3	18.7
4		Cd_6_Gd	*a *= 15.5253(6)	85.4	14.6
Cd_83.5_Gd_16.5_	600; 2 months; 600	Cd_45_Gd_11_	*a = *21.6076(9)	–a	–a
		Cd_58_Gd_13_	*a *= 15.3963(6), *c *= 15.2661(1)		
5	500; 3 months; 500	Cd_6_Gd	*a *= 15.5265(3)	–b	–b
Cd_82.3_Gd_17.7_		Cd_58_Gd_13_	*a *= 15.3969(4), *c *= 15.2659(7)		
6	700; 3 months; 900	Cd_45_Gd_11_	*a = *21.5964(1)	80.9	19.1
Cd_80.6_Gd_19.4_					
7	750; 3 months; 950	Cd_45_Gd_11_	*a *= 21.6058(3)	79.8	20.2
Cd_79_Gd_21_		Cd_3_Gd	*a = *6.6272(4), *c *= 4.9359(6)	74.9	25.1
8	700; 3 months; 950	Cd_45_Gd_11_	*a *= 21.6091(2)	79.9	20.1
Cd_78_Gd_22_		Cd_3_Gd	*a = *6.6228(4), *c *= 4.9329(6)	75.0	25.0
9	750; 4 months; 950	Cd_3_Gd	*a = *6.6194(6), *c *= 4.9337(8)	74.9	25.1
Cd_73_Gd_27_		α-Cd_2_Gd	*a = *4.9404(6), *c *= 3.4711(5)	67.2	32.8
10	700; 3 months; 1100	Cd_3_Gd	*a = *6.6220(4), *c *= 4.9342(6)	74.8	25.2
Cd_71.2_Gd_28.8_		α-Cd_2_Gd	*a = *4.9403(2), *c *= 3.4673(2)	67.1	32.9
11	750; 3 months; 900	Cd_3_Gd	*a = *6.6090(1), *c *= 4.9389(2)	74.8	25.2
Cd_69_Gd_31_		α-Cd_2_Gd	*a = *4.9407(3), *c *= 3.4669(3)	67.5	32.5
12	750; 4 months; 1050	α-Cd_2_Gd	*a = *4.9432(2), *c *= 3.4698(2)	–c	–c
Cd_63_Gd_37_		CdGd	*a *= 3.7501(8)	50.8	49.2
13	950; 4 months; 1050	α-Cd_2_Gd	*a = *4.9406(2), *c *= 3.4702(3)	66.9	33.1
Cd_63_Gd_37_		CdGd	*a *= 3.7487(1)	51.0	49.0
14	900; 2 months; 1200	α-Cd_2_Gd	*a = *4.9406(2), *c *= 3.4657(2)	66.6	33.4
Cd_58.8_Gd_41.2_		CdGd	*a *= 3.7482(3)	51.1	48.9
15	1003; 2 months; 1200	α-Cd_2_Gd	*a = *4.9432(3), *c *= 3.4667(3)	66.4	33.6
Cd_58.8_Gd_41.2_		CdGd	*a *= 3.7470(3)	51.3	48.7
16	950; 3 months; 1200	α-Cd_2_Gd	*a = *4.9410(2), *c *= 3.4715(3)	66.8	33.2
Cd_54_Gd_46_		CdGd	*a *= 3.7470(5)	51.3	48.7
17	850; 3 months; 1200	CdGd	*a *= 3.7573(5)	49.3	50.7
Cd_45_Gd_55_		β-Gd	–	21.2	78.8
18	700; 2 months; 1200	CdGd	*a *= 3.7596(3)	49.5	50.5
Cd_40_Gd_60_		α-Gd	*a = *3.642(3), *c *= 5.7602(8)	3.0	97.0
19	800; 3 months; 1200	CdGd	*a *= 3.7561(4)	49.5	50.5
Cd_40_Gd_60_		β-Gd	–	20.5	79.5
20	900; 2 months; 1200	CdGd	*a *= 3.7554(3)	49.1	50.9
Cd_40_Gd_60_		β-Gd	–	22.6	77.4
21	650; 3 months; 1200	CdGd	*Too ductile*	49.5	50.5
Cd_35_Gd_65_		α-Gd		2.8	97.2
22	600; 3 months; 1200	CdGd	*Too ductile*	49.8	50.2
Cd_30_Gd_70_		α-Gd		1.9	98.1
23	850; 3 months; 1200	CdGd	*Too ductile*	49.5	50.5
Cd_25_Gd_75_		β-Gd		21.2	78.8
24	750; 3 months; 1200	CdGd	*Too ductile*	49.8	50.2
Cd_22_Gd_78_		β-Gd		19.1	80.9
25	900; 3 months; 1200	β-Gd	*Too ductile*	17.3	82.7
Cd_17_Gd_83_					
26	800; 2 months; 1200	β-Gd	*Too ductile*	16.9	83.1
Cd_10_Gd_90_		α-Gd		4.3	95.7
27	900; 2 months; 1200	β-Gd	*Too ductile*	–c	–c
Cd_10_Gd_90_		α-Gd		4.4	95.6

a Sample not in equilibrium; EDX data of Cd_45_Gd_11_ and Cd_58_Gd_13_ could not be separated, see Section 3.2.

b Sample, taken from isopiestic measurements [13], was too brittle to prepare for EDX.

c Microstructure of the respective phase was too fine to measure accurately with EDX.

After pre-heating of the samples to a temperature just above the melting point of Cd, they were homogenised at elevated temperatures, specified as *T*_max_ in Table 2, and further annealed. Samples with a Cd content less than 22 at.% were homogenised at 1400 °C using an induction furnace. At this temperature Cd is already gaseous which could cause a violent pressure increase inside the crucibles. Thus a reliable homogenisation was only expected when Cd had already completely reacted with Gd during the pre-heating step. It turned out that samples with higher amounts of Cd than 22 at.% led to severe expansion of the crucibles when heated above 1200 °C. Hence, these samples were slowly heated (0.5 K/min) to temperatures were the corresponding alloy was completely or partially melted. After pre-heating and homogenisation at elevated temperatures, the samples were annealed for at least five weeks at different temperatures (Table 2) and subsequently quenched in cold water.

Phase identification and precise lattice parameters of the different phases were obtained by means of powder X-ray diffraction (powder-XRD) using a Bruker D8 Discover Series 2 powder diffractometer in Bragg–Brentano pseudo-focusing geometry and employing Cu Kα radiation. Data were collected by a LynxEye silicon strip detector (exposure time: 2 h). Additional measurements were performed on a Guinier–Huber camera 670 operating with Cu Kα_1_ radiation and an image plate detector (measurement period: 2 h, 10 detection loops). Special sample holders with X-ray transparent lids were used to prevent the sample powders from oxidation. The corresponding powder-XRD patterns were analysed and refined by means of the TOPAS 3 software, applying the fundamental parameter approach for peak profile modelling.

For investigation of the microstructures, selected samples were embedded in phenolic hot mounting resin and then ground and polished. Grinding was carried out with silicon carbide abrasive paper (mesh size: 400, 600, 800, 1000 and 1200) using water as fluid. Although Gd containing alloys are essentially sensitive to water, it was sufficient to keep the grinding steps as short as possible to prevent oxidation. Grinded samples were intermediately stored in cyclohexane. For fine polishing of the surface a water-free diamond suspension was taken with kerosene as fluid.

Metallographic analyses were carried out on a binocular reflected-light microscope (Zeiss Axiotech 100) featured with a bi-refringent prism for differential interference contrast (DIC) imaging and equipment for operation under polarized light. Quantitative examinations of the microstructures were performed on a Zeiss Supra 55 VP environmental scanning electron microscope (SEM) using pure elements as standard materials and Co for the energy calibration of the energy-dispersive X-ray (EDX) detector signal. A 120 μm aperture was used and an acceleration voltage of 20 kV was applied. For imaging of the microstructures, a back-scatter detector was employed. Final compositions were calculated by conventional ZAF matrix correction from the measured X-ray intensities. The composition of each phase was measured at ten or more spots in order to minimize statistical errors and to obtain more reliable results.

Differential thermal analysis (DTA) was carried out on a Netzsch DSC 404 F1 Pegasus using closed Ta crucibles with flat bottoms and a constant Ar flow of 50 mL/min, respectively. Sapphire was used as the reference material. The temperature was measured with type-S (Pt/Pt10%Rh) thermocouples which were calibrated at the melting points of the high purity metals Sn, Al, Ag and Cu. The samples weighed usually around 150–200 mg and were positioned in good thermal contact to the crucibles. Optionally, samples were powdered and pressed into pills to ensure good contact of the individual grains for better diffusion. Two heating- and cooling-curves were recorded for each sample using a heating rate of 5 K min^−1^.

## 3 Results and discussions

A number of samples were annealed and characterised by powder-XRD, SEM and DTA, to reinvestigate the equilibrium phase diagram of Cd–Gd, presented first by Bruzzone et al. [14]. In particular, it was of interest to verify the homogeneity ranges of the intermetallic compounds (see below) which were recently derived from isopiestic vapour pressure measurements [13]. Moreover, all phase equilibria and the complete reaction scheme were re-examined and discussed. A selection of relevant samples, examined with isothermal methods, is listed in Table 2. Heat treatments, identified phases and phase compositions are given.

**Fig. 1:**
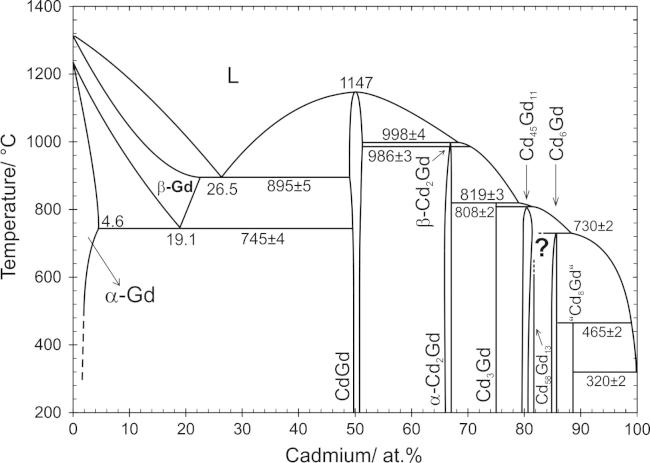
Cd–Gd phase diagram according to the present results. The solubility of Gd in liquid Cd between 324 and 500 °C was taken from Johnson [17].

All samples which were studied by DTA are listed in Table 3, together with their thermal effects from two heating and cooling cycles, respectively. On the basis of the combined results a complete version of the Cd–Gd phase diagram was drawn, which is shown in Fig. 1.

**Table 3:** Thermal effects of selected samples determined with DTA.

Sample	Nominal comp. (at.%)	Annealing temperature (°C)	Heating (°C)					Cooling (°C)
			Invariant effects		Other effects		Liquidus	Liquidus
1	Cd_98_Gd_2_	300	319				–	526
2	Cd_90_Gd_10_	300	321				723	700
3	Cd_83.5_Gd_16.7_	700	730				798	789
6	Cd_80.6_Gd_19.4_	700	808				813	806
7	Cd_79_Gd_21_	750	808				814	807
8	Cd_78_Gd_22_	700	808	822			841	831
9	Cd_73_Gd_27_	750	819				990	945
10	Cd_71.2_Gd_28.8_	700	821				986	969
11	Cd_69_Gd_31_	750	816	–a			991	988
12, 13	Cd_63_Gd_37_	750, 950	985				1055	–
14, 15	Cd_58.8_Gd_41.2_	900, 1003	988	998			1093	1068
16	Cd_54_Gd_46_	950	984	–b			1128	1036
17	Cd_45_Gd_55_	850	887				1133	1125
18, 19, 20	Cd_40_Gd_60_	700, 800, 900	746	900			1097	1096
21	Cd_35_Gd_65_	650	746	–b			1047	1042
22	Cd_30_Gd_70_	600	745	898			967	966
23	Cd_25_Gd_75_	850	–b				936	913
24	Cd_22_Gd_78_	750	742				976	976
25	Cd_17_Gd_83_	900	746		782	931	1071	1062
26, 27	Cd_10_Gd_90_	800, 900	746		942	1057	1202	1202
28	Cd_96_Gd_4_	300	320				639	578
29	Cd_95_Gd_5_	300	320				660	658
30	Cd_92_Gd_8_	300	318				707	686
31	Cd_88.7_Gd_11.3_	350	465				727	727
32	Cd_77.6_Gd_22.4_	700	810	821			840	811
33	Cd_50_Gd_50_	750					1147	1132
34	Cd_16_Gd_84_	900	745		795	942	1080	1080
35	Cd_15_Gd_85_	700	746		784	945	1081	1081
36	Cd_6_Gd_94_	700	743				1233	1233
37	Cd_3_Gd_97_	700					1282	1280

a DTA curve shows an invariant effect in the cooling curve probably caused by the polymorphic transformation of Cd_2_Gd.

b The onset could not be evaluated due to an abnormal peak shape; invariant effects can be clearly seen in the cooling curves.

### 3.1 Phase equilibria, reaction scheme and a new compound “Cd_8_Gd”

As can be seen in Table 2, several samples were annealed at different temperatures to accurately determine the homogeneity ranges of the corresponding intermetallic compounds. Unfortunately, it was not possible to examine all samples with both methods, i.e., SEM and powder-XRD. Samples containing large amounts of Cd or Gd, respectively, turned out to be rather ductile; consequently powdering of these samples was virtually impossible. Attempts to grind samples with a Gd content higher than 60 at.% into powders failed: apart from the fact that they were very ductile, Gd has a high atomic number and thus absorption effects were found to be quite high when irradiated by X-rays. The resulting low intensities made it impossible to perform an accurate evaluation of the lattice parameters of α- and β-Gd. With respect to its ductility, no powder-XRD was carried out for sample 1 as well. Indeed, the lattice parameters of Cd were assumed to be similar to those from sample 2, which was annealed at 300 °C.

The overall composition of each sample, obtained by EDX, was compared with its nominal composition to estimate possible weight losses due to volatile Cd. No significant deviation of the determined composition from the nominal composition was found in most of the samples; e.g., nominal compositions of the single-phase samples 6 and 25 (Table 2) are in excellent agreement with EDX results. Even for samples with high Cd contents only a very thin Cd film could be observed visually inside the crucibles indicating negligible deviation from the nominal composition. As all homogeneity ranges were derived from several samples and most of them were prepared twice, an experimental error of about ±0.5 at.% for EDX measurements could be estimated. From the comparison of the EDX results of the stoichiometric compound Cd_3_Gd it can be clearly seen that all values lie within the estimated error limit, see Table 4.

**Table 4:** Estimated phase boundaries of Cd–Gd phases at 500 °C (EDX) together with corresponding melting or decomposition temperatures averaged from DTA results.

Phase	Phase boundaries (at.% Cd)	Melting or decomposition temperatures (°C)
“Cd_8_Gd“	88.7 (line compound)	465
Cd_6_Gd	84.8–85.7	730
Cd_58_Gd_13_	See text	
Cd_45_Gd_11_	79.6–80.8	808
Cd_3_Gd	75.0 (line compound)	819
Cd_2_Gd	65.8–67.1	998^a^
CdGd	49.8–50.9	1147

a Cd_2_Gd transforms into a high-temperature modification at 986 °C.

EDX measurements of samples 1 and 2 indicated a new intermetallic compound with the stoichiometric composition Cd_88.7_Gd_11.3_. Corresponding powder-XRD results as well as the formation behaviour of this new compound, which corresponds to the empirical formula “Cd_8_Gd”, are described below. Unfortunately, it was not possible to obtain structural data of “Cd_8_Gd” in the present study. Work is still going on to produce suitable single crystals for a proper structure determination.

As pointed out, homogeneity ranges of all intermetallic compounds were defined at 500 °C and are listed in Table 4. Apparently, these values agree quite well with phase boundaries reported recently [13] and listed in Table 1. The present data indicate narrow but finite homogeneity ranges for all compounds except Cd_3_Gd and “Cd_8_Gd”, which were identified as line compound, i.e., no significant solid solubility of either Cd or Gd was determined. The largest deviation of the solubility limits was observed for the Gd-rich boundary of CdGd. The homogeneity ranges given in Ref. [13] are based on powder-XRD results of equilibrated CdGd alloys. It is possible that, due to the discussed absorption effects of Gd, samples were misinterpreted to be phase-pure CdGd although they might actually have been located already in the two-phase field α-Gd + CdGd. Thus, the solubility of Gd within CdGd at 500 °C is probably less than indicated in Ref. [13] and much closer to the value obtained here.

All binary phase reactions were examined by means of DTA. For the description of the reaction scheme and the graphical representation of the phase diagram (Fig. 1), samples listed in Table 2 as well as additional equilibrated samples were taken. Effects measured from samples with identical nominal compositions were usually averaged. The corresponding results of the DTA measurements of all samples are listed in Table 3. For each sample two heating and cooling cycles were performed at a heating rate of 5 K/min in order to check if equilibrium conditions can be restored after the first melting of the sample. It was found that most of the alloys were still in equilibrium after the first cycle but, for the graphical representation of the phase diagram, all thermal effects, except the liquidus, were taken from the first heating curves. In case of the liquidus effect, usually an average value from the two heating curves was taken for the evaluation. Although most of the cooling curves exhibited considerable supercooling, their liquidus values were still considered to guide the construction of the liquidus curves.

In addition to the new compound “Cd_8_Gd”, all six intermetallic compounds, which were already described by Bruzzone et al. [14], were confirmed in the current study. All invariant reactions, evaluated from the present results, are listed in Table 5 together with the respective reaction temperatures and types and the phase compositions. As can be seen, all compounds except CdGd are formed through a cascade of incongruent reactions which was also obtained by Bruzzone et al. Although the reaction scheme is in agreement with that of the latter authors, there are some discrepancies with regard to the isothermal reaction temperatures and the liquidus shape. In addition, the homogeneity ranges of the low- and high-temperature modifications of Gd seem to be narrower than indicated by Ref. [14]. Distinct differences are discussed below in Section 3.3. To clarify the formation reactions of the two intermetallic compounds Cd_45_Gd_11_ and Cd_58_Gd_13_, with compositions rather close to each other, the composition range between 78 and 82 at.% Cd was extensively investigated (see Section 3.2.).

**Table 5:** Invariant reactions in the system Cd–Gd derived from a combination of all present results.

Reaction	*T*/°C	Phase compositions (at.% Cd)			Reaction type
L ⇄ Cd + Cd_8_Gd	320 ± 2	99.8^a^	∼100	88.7	Degenerate eutectic
L + Cd_6_Gd ⇄ Cd_8_Gd	465 ± 2	99.0^a^	85.7	88.7	Peritectic
L + Cd_45_Gd_11_ ⇄ Cd_6_Gd	730 ± 2	88.2	81.7	85.7	Peritectic
Cd_6_Gd + Cd_45_Gd_11_ ⇄ Cd_58_Gd_13_	–b				Probably peritectoid
L + Cd_3_Gd ⇄ Cd_45_Gd_11_	808 ± 2	81.5	75.0	80.2	Peritectic
L + α-Cd_2_Gd ⇄ Cd_3_Gd	819 ± 3	79.0	67.1	75.0	Peritectic
L + CdGd ⇄ β-Cd_2_Gd	998 ± 4	68.3	51.2	66.1	Peritectic
α-Cd_2_Gd ⇄ β-Cd_2_Gd	986 ± 3				Polymorphic transformation
CdGd ⇄ L	1147 ± 5		50.0		Congruent melting
L ⇄ CdGd + β-Gd	895 ± 5	26.5	49.0	22.6	Eutectic
β-Gd ⇄ CdGd + α-Gd	745 ± 4	19.1	49.6	4.6	Eutectoid

a Solubility values were taken from Johnson et al. [17].

b Isothermal reaction temperature could not be defined according to the present results, compare 3.2.

In the very Cd-rich part of the phase diagram, Bruzzone et al. described a eutectic reaction L ⇄ Cd_6_Gd + Cd at 97.5 at.% Cd. The rather extended solubility of Gd in liquid Cd was extrapolated from DTA results but does not correspond to data presented earlier by Johnson [17]. Johnson determined much smaller solubilities, i.e., varying from 0.17 to 1.45 at.% Gd between 324 and 500 °C. These values are in excellent agreement with the present DTA data of samples with nominal compositions between 90 and 98 at.% Cd (Table 3). Thus the liquidus curve between 98 and 100 at.% Cd (Fig. 1) was fitted according to Johnson’s solubility data which result apparently in a degenerate eutectic reaction, i.e., L ⇄ Cd_8_Gd + Cd.

**Fig. 2:**
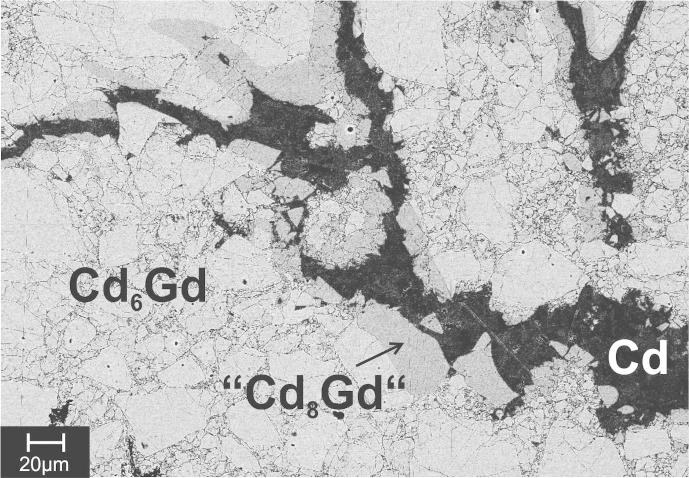
Microstructure (BSE image) of an alloy with the nominal composition Cd_90_Gd_10_ (No. 2, Table 2).

Several alloys were prepared to produce an almost single phase sample of “Cd_8_Gd”. It turned out that this phase forms rather slowly, especially when heating the respective alloy above its overall melting point. An alloy with the nominal composition Cd_90_Gd_10_ (No. 2, Table 2) was homogenised at 800 °C before it was annealed for about 2 month at 300 °C. Its corresponding BSE image is shown in Fig. 2. Only a small amount of “Cd_8_Gd” was found to be present, exclusively occurring at the boundaries between Cd and Cd_6_Gd grains. This typical microstructure argues for a peritectic formation of “Cd_8_Gd”. Samples produced in the same way as sample 2 but with higher Cd contents i.e., 92, 96 and 98 at.%, were produced in order to achieve equilibrium alloys within the two-phase field Cd + “Cd_8_Gd”. It turned out that with increasing Cd contents of the alloys and a corresponding lower melting point, the amounts of Cd_6_Gd could be decreased and an equilibrium sample within the two-phase field Cd + “Cd_8_Gd” could be prepared (No. 1, Table 2). This is probably related to shorter diffusion paths due to smaller primary crystals of Cd_6_Gd. It seems that the peritectic formation of “Cd_8_Gd” is rather determined kinetically than thermodynamically. As a consequence, all attempts to produce a single-phase sample of “Cd_8_Gd” by annealing an as-cast alloy failed.

**Fig. 3:**
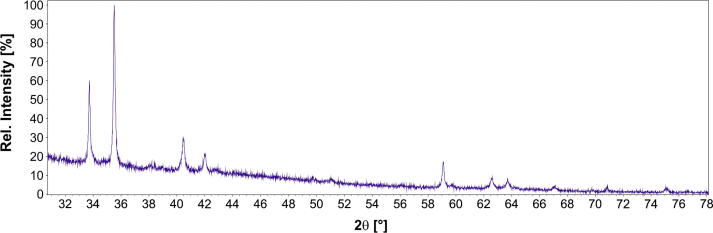
Powder-XRD pattern of a single-phase alloy with the nominal composition Cd_88.7_Gd_11.3_ which corresponds to “Cd_8_Gd”.

Therefore, a sample with the nominal composition Cd_88.7_Gd_11.3_, was slowly heated to 350 °C and held at this temperature for 2 months without prior homogenisation at elevated temperatures. In this case, Gd powder was used to achieve a better mixing of the metals. The corresponding BSE image showed that this alloy was phase-pure “Cd_8_Gd” with the measured composition Cd_88.6_Gd_11.4_. The respective powder-XRD pattern, given in Fig. 3, shows likewise that this alloy is phase-pure and no additional reflections belonging to Cd and/or Cd_6_Gd were obtained. It is assumed that after the melting point of Cd was reached, initially the phase richest in Cd (“Cd_8_Gd”) was formed at the surface boundaries of the Gd particles. Probably, these surface layers acted as nuclei which actually promoted the formation of “Cd_8_Gd”.

**Fig. 4:**
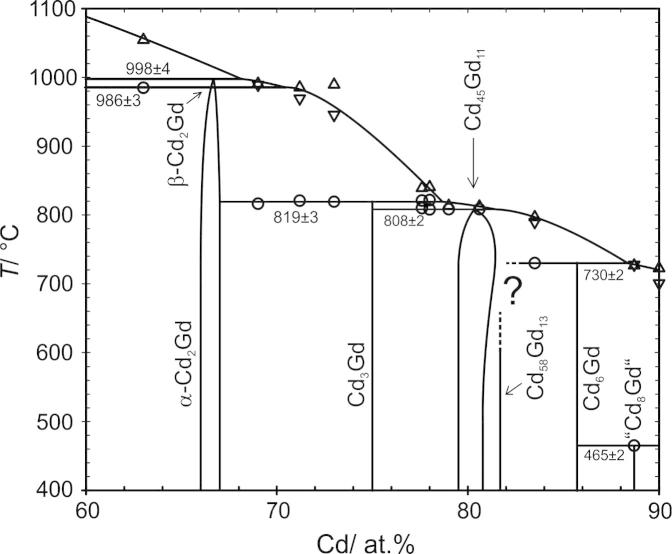
Partial phase diagram of Cd–Gd between 60 and 90 at.% Cd. Large circles: invariant thermal effects. Triangles up: liquidus on heating. Triangles down: liquidus on cooling.

As already observed by Bruzzone et al. [14], the present DTA results indicated a polymorphic transformation of Cd_2_Gd into a high-temperature modification β-Cd_2_Gd. Its corresponding transition temperature as well as the peritectic decomposition temperature of β-Cd_2_Gd were slightly lower than given in Ref. [14], i.e., 986 and 998 °C instead of 995 and 1010 °C, see Fig. 4. Although these two invariant reactions were clearly indicated in all DTA curves of corresponding samples, it was often not possible to evaluate the peritectic decomposition temperature precisely, especially when both effects strongly overlapped. Several attempts were carried out to quench the high-temperature modification of β-Cd_2_Gd but were not successful. Efforts are still going on to clarify its structure.

**Fig. 5:**
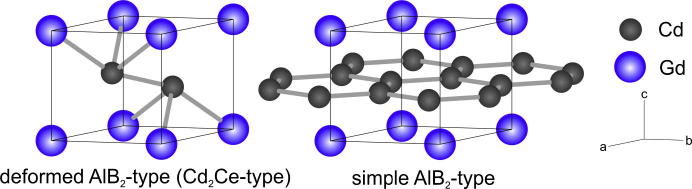
Comparison of the deformed (Cd_2_Ce-type, $P\overset{¯}{3}m1$) and the simple AlB_2_-type (*P*6/*mmm*) structure.

Iandelli and Palenzona [29] as well as Mulokozi [32] described α-Cd_2_Gd to crystallize in the Cd_2_Ce-type, a deformed AlB_2_-type structure with space group symmetry $P\overset{¯}{3}m1$, where Cd occupies the site 2(*d*) (1/3 2/3 *z*) with *z* = 0.42. The respective unit cell is shown in Fig. 5. More recently, a single-crystal investigation of iso-structural α-Cd_2_Pr [10] showed that this phase crystallizes with space group *P*6/*mmm* and the corresponding site positions were determined at (1/3 2/3 1/2) and (0 0 0). The latter structure is currently known as the simple AlB_2_-type (Fig. 5). Compared to the Cd_2_Ce-type, there is a planar arrangement of Cd atoms with equidistant spacing to all neighbouring Gd atoms. This ordering effect leads to an increase of the cell symmetry and a change from space group $P\overset{¯}{3}m1$ to its supergroup *P*6/*mmm* according to the group/subgroup relation. As a consequence, the calculated powder-XRD patterns of Cd_2_Ce and AlB_2_ look slightly different. It was observed that especially the reflexes (0 1 2) and (2 1 2) seem to be systematically extinguished in the AlB_2_-type, a fact that corresponds to the present observed powder-patterns of α-Cd_2_Gd. Therefore, it is assumed that α-Cd_2_Gd is also crystallizing with space group *P*6/*mmm* and the corresponding Cd position (1/3 2/3 1/2) as listed in Table 1.

### 3.2 The formation behaviour of Cd_45_Gd_11_ and Cd_58_Gd_13_

As previously indicated by Bruzzone et al. [14], two compounds are located in the rather narrow composition range 80–82 at.% Cd, namely Cd_58_Gd_13_ and Cd_45_Gd_11_. Compounds with this stoichiometry were found to be in thermodynamic equilibrium in a number of Cd-RE phase diagrams but Cd–Gd is the only system were both phases decompose within a rather close temperature interval, i.e., 803 and 806 °C [14]. Hence, these phases should be quite similar with respect to their relative stability. The existence of a narrow but definite two-phase field along the entire temperature interval indicates that they are truly separate phases and not just parts of a solid solution. Actually, this was not to be expected because there is no structural relation between the crystal structures of Cd_58_Gd_13_ and Cd_45_Gd_11_. In order to elucidate the temperatures and the sequence of the corresponding formation reactions it was tried to prepare a two-phase alloy of Cd_58_Gd_13_ and Cd_45_Gd_11_.

Initially, a sample, with a nominal composition located within the two-phase field of Cd_58_Gd_13_ and Cd_45_Gd_11_ (No. 6, Table 2), was annealed at 700 °C. As can be seen from the EDX and powder-XRD results in Table 2, this sample was a single-phase alloy of Cd_45_Gd_11_. Next, a sample with the nominal composition Cd_83.5_Gd_16.5_ (No. 3) was annealed at 700 °C. Differently than expected from the phase diagram [14], a two-phase equilibrium between Cd_6_Gd and Cd_45_Gd_11_ was obtained for this alloy. All further attempts to produce an alloy, which contains Cd_58_Gd_13_ at temperatures above 700 °C failed. In fact, all respective samples were observed to be two-phase alloys of Cd_6_Gd and Cd_45_Gd_11_. Based on the EDX results of sample No. 3, the homogeneity range of Cd_45_Gd_11_ could be limited at a composition of 81.3 at.% Cd at 700 °C (Fig. 4). This relatively high solubility of Cd within Cd_45_Gd_11_ at 700 °C explained why sample 6 was just single-phase.

Since there was no doubt that Cd_58_Gd_13_ is an equilibrium phase, which was recently found in isopiestic samples between 430 and 530 °C [13], it was assumed that it decomposes at some temperature below 700 °C. Therefore, a sample (No. 4), with the nominal composition equal to sample No. 3, was annealed at 600 °C without prior homogenisation at elevated temperatures. The corresponding powder-XRD pattern clearly showed presence of Cd_58_Gd_13_ with comparable amounts of its neighbouring phases Cd_6_Gd and Cd_45_Gd_11_, respectively. Although this sample was not in equilibrium, it indicated that Cd_58_Gd_13_ is an equilibrium phase. Nevertheless, all attempts to prepare an equilibrium sample, which contains Cd_58_Gd_13_ only, were not successful.

**Fig. 6:**
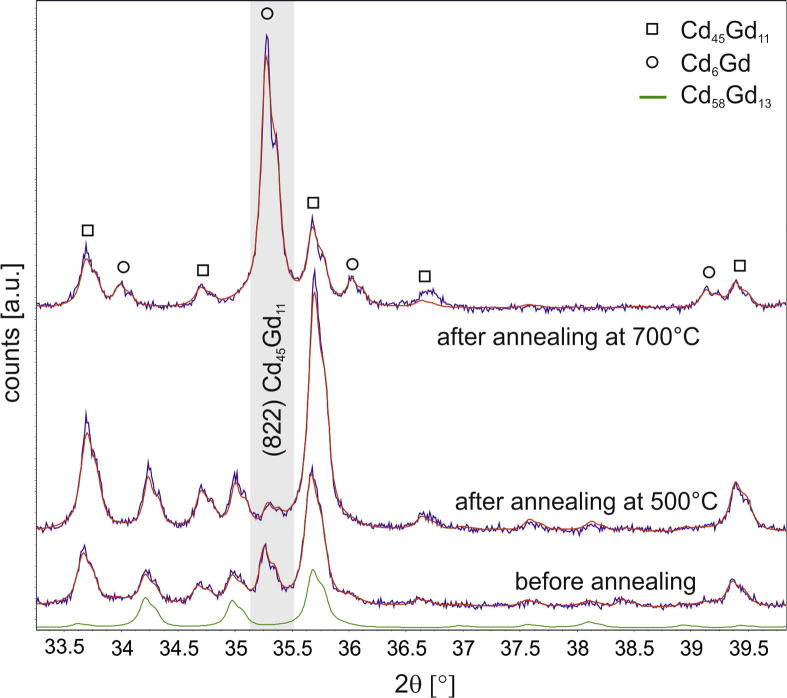
Powder-XRD patterns of alloys, with the nominal composition Cd_82.3_Gd_17.7_, before as well as after annealing at 500 and 700 °C. Reflex (822) indicates the relative increase and decrease of Cd_45_Gd_11_.

Therefore, seven well equilibrated samples from the isopiestic vapour pressure measurements (see Ref. [13]; run/sample: 1/1, 3/1, 6/2, 6/3, 7/3–7/5), which contained considerable amounts of Cd_58_Gd_13_, were powdered and pressed into pills, which allowed for rather short diffusion paths. It was assumed that initially present Cd_58_Gd_13_ crystals would promote its further growth. The overall composition of the respective pills was Cd_82.3_Gd_17.7_, which is rather close to the stoichiometric composition of Cd_58_Gd_13_. The pills were annealed for 3 months at 500 and 700 °C and the corresponding powder-XRD patterns before as well as after annealing are shown in Fig. 6. Before annealing, the alloy contained comparable amounts of Cd_6_Gd, Cd_58_Gd_13_ and Cd_45_Gd_11_. It was observed that after annealing at 500 °C, the amount of Cd_45_Gd_11_ had considerably decreased by forming Cd_6_Gd and Cd_58_Gd_13_. On the contrary, the alloy which was annealed at 700 °C showed a noticeable increase of Cd_45_Gd_11_ whereas Cd_58_Gd_13_ had completely disappeared. This behaviour agrees well with the results described above. In addition, all samples, which contained Cd_58_Gd_13_, were investigated by DTA in order to obtain the decomposition temperature of this compound. Unfortunately, no additional effect, apart from the effects dedicated to the decomposition reactions of Cd_6_Gd and Cd_45_Gd_11_, was observed in the respective heating curves. However, some of the cooling curves comprised an additional peak just below the melting effect. Thus, powder-XRD measurements were performed from DTA samples after the final cooling run, in order to see if Cd_58_Gd_13_ was formed. Unfortunately, no Cd_58_Gd_13_ was in any of these alloys.

As a conclusion, it has to be assumed that Cd_58_Gd_13_ is not stable at temperatures above 700 °C. Obviously, this phase is formed through a peritectoidic reaction between Cd_6_Gd and Cd_45_Gd_11_, *cf*. Table 5. Attempts are still going on to clarify the range which is currently labelled with a question mark in Fig. 4.

**Fig. 7:**
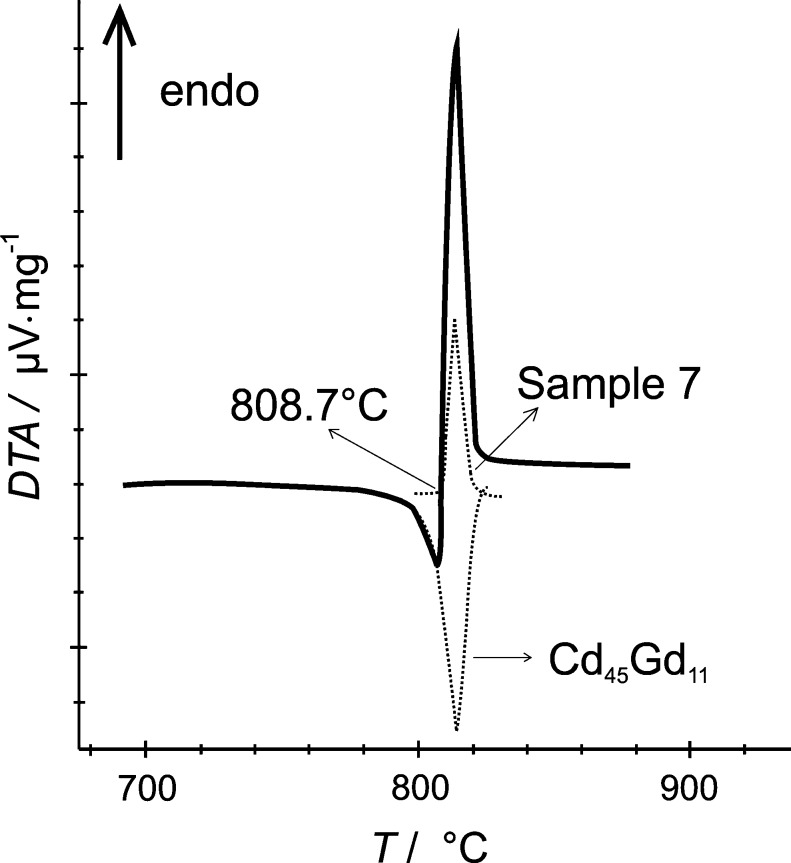
DTA curve of sample 7 against pure Cd_45_Gd_11_ as reference. Corresponding DTA results from sample 7 and Cd_45_Gd_11_ against NIST standard sapphire as reference are shown as dotted curves for comparison.

To confirm the incongruent formation of Cd_45_Gd_11_, an alloy with the respective stoichiometric composition was prepared by equilibration with Cd vapour as in the isopiestic vapour pressure measurements [13]; powder-XRD confirmed that this alloy was phase-pure Cd_45_Gd_11_. Since the liquidus temperature of this alloy seems to be rather close to the peritectic decomposition temperature of Cd_45_Gd_11_ [14], no clear proof for an incongruent melting behaviour could be obtained by DTA. Actually, the DTA curve looked quite similar to that of a congruently melting phase. Hence, a sample with the nominal composition Cd_79_Gd_21_ (No. 7) was measured in DTA against pure Cd_45_Gd_11_ as the reference material. It was assumed that both sample and reference position are symmetrical within the furnace. If Cd_45_Gd_11_ were a congruently melting phase, one would first expect a eutectic effect caused by the two-phase sample, followed by the congruent melting effect of the Cd_45_Gd_11_ reference. Contrary, if Cd_45_Gd_11_ is an incongruently melting phase, the trend of the resulting DTA curve would be strongly determined from the difference in mass of the sample and the reference. The corresponding DTA curve is shown in Fig. 7, superimposed onto the DTA curves from sample No. 7 and Cd_45_Gd_11_ against NIST^1^ standard sapphire as reference material. It can be seen, that there is an initial hypothetically exothermal effect which belongs to a reaction within the reference material, i.e., Cd_45_Gd_11_. Apparently, Cd_45_Gd_11_ has to be an incongruently melting phase because otherwise one would definitely expect an endothermic effect at first. It can even be observed, that this effect follows the peak which was observed in the DTA curve from pure Cd_45_Gd_11_ against NIST sapphire. The evaluation of the onset of the “exothermal” effect resulted in a value of 806.0 °C which is taken as the peritectic reaction temperature of Cd_45_Gd_11_. The onset of sample No. 7 also corresponds to the peritectic formation of Cd_45_Gd_11_ but since it is more distinct, the temperature value is much closer to the average reaction temperature of 808 ± 2 °C (*cf*. Table 5).

### 3.3 The composition range 0–52 at.% Cd

**Fig. 8:**
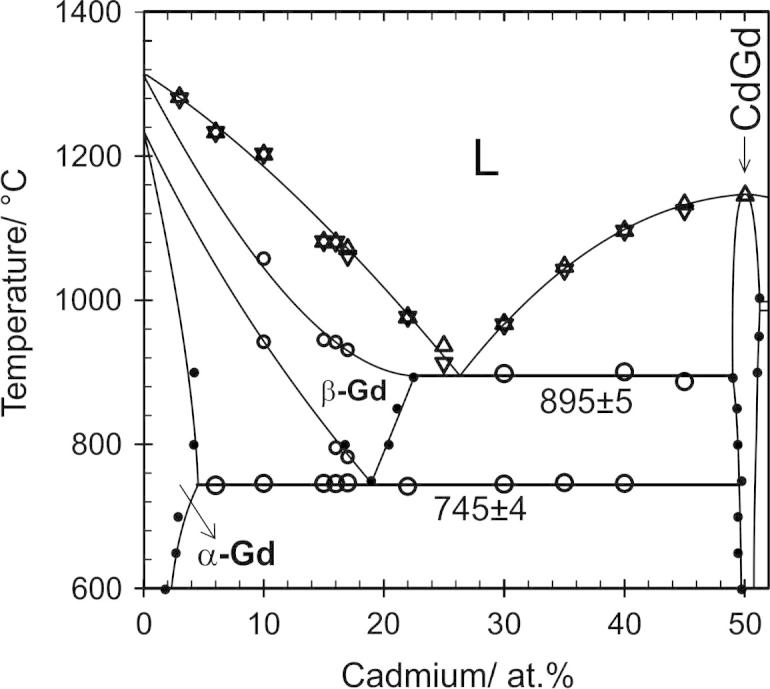
Partial phase diagram of Cd–Gd between 0 and 52 at.% Cd. Large circles: invariant thermal effects. Small open circles: non-invariant effects. Triangles up: liquidus on heating. Triangles down: liquidus on cooling. Small filled circles: EDX data.

The partial equilibrium phase diagram between 0 and 52 at.% Cd is shown in Fig. 8 together with the experimental data points from DTA and EDX measurements (Table 2, Table 3). Although the reaction scheme of Bruzzone et al. [14] could be confirmed, isothermal reaction temperatures as well as homogeneity ranges of the low- and high-temperature modifications of Gd differ noticeably. The melting point of CdGd was determined to be about 23 °C lower, namely 1147 °C (Table 3). The isothermal reaction temperature of the eutectic reaction L ⇄ CdGd + β-Gd was obtained to be 895 ± 5 °C. Actually, this value is about 25 °C lower than given in Ref. [14] and clearly disagrees from the estimation by Gschneidner and Calderwood [20], see Section 1. However, the corresponding eutectic point was found to be quite similar to that given earlier, i.e., 26.5 instead of 27.0 at.% Cd [14].

Veleckis and Van Deventer [46] listed isothermal reaction temperatures for eutectic reactions between RE elements and the respective compound richest in RE, which is, with the exception of Cd_5_Eu_6_, CsCl-structured CdRE. As indicated by Gschneidner and Calderwood [24] the eutectic temperatures do not fit well with subsequently reported values. The largest differences between values given by Veleckis and Van Deventer and those reported later occurred when the corresponding RE undergoes an allotropic transformation. It has to be assumed that Veleckis and Van Deventer did not consider the high-temperature allotropic modifications of the respective RE elements. Indeed, their reported value for the eutectic reaction at 719 °C corresponds reasonably well with the present value for the eutectoid decomposition of β-Gd, namely 745 ± 4 °C. This latter value was averaged from DTA data of nine samples, see Fig. 8. Actually, the eutectoid reaction temperature was altered a few times in literature. First given as 725 °C by Bruzzone et al., it was more recently determined by Tang and Gschneidner [21] to be about 738 °C. Tang and Gschneidner performed DTA measurements with a heating rate of 2 K/min whereas in the present study 5 K/min was used. According to the deviation of the measured transition temperatures, it has to be assumed that the transformation from α- to β-Gd is kinetically determined. In fact, β-Gd was sometimes found in samples annealed below the eutectoid temperature. By extrapolation of the heating rate to 0 K/min, the eutectoid reaction temperature is obtained to be 734 °C, a temperature which is probably closer to the equilibrium transformation temperature.

The respective DTA results fit excellently with phase compositions obtained from EDX measurements. Hence, the homogeneity ranges of α- and β-Gd could be determined rather accurately. The maximum solubility of Cd in β-Gd was determined as 22.6 at.% (*cf.* sample No. 20). This corresponds reasonably with data from Bruzzone et al. [14] whereas the shape of the phase boundaries of β-Gd is somewhat different from those in Ref. [14]. Since the eutectoid decomposition point could be defined reliably by EDX at Cd_19.1_Gd_80.9_, this unusual phase boundary is apparent.

**Fig. 9:**
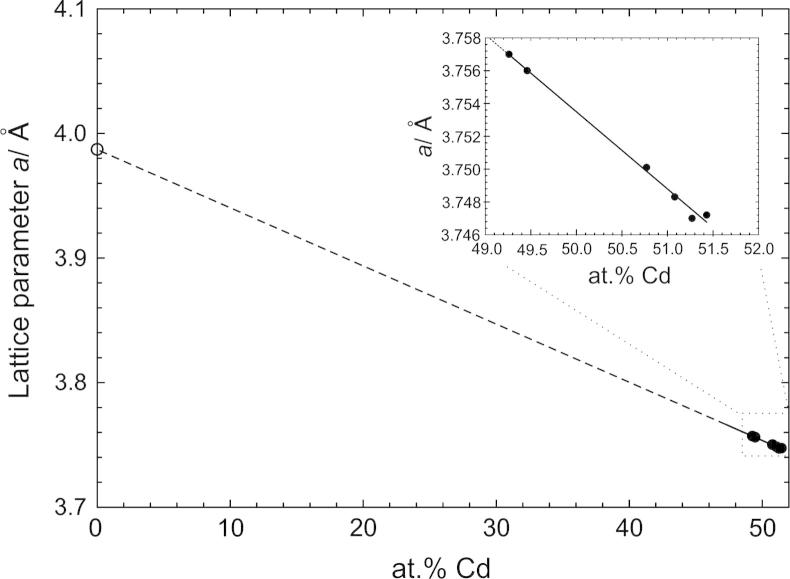
Lattice parameter *a* against at.% Cd for CdGd; filled circles: sample compositions defined by EDX, see Section 3.3.

The compound richest in Gd is CdGd. It crystallizes in the CsCl-structure type and exhibits an ordered variety of the β-Gd structure which crystallizes in the *bcc* W-structure (*a* = 3.99 ± 0.04 Å [21]); half of the Gd sites in β-Gd are substituted by Cd atoms. Besides, a partial substitution of Cd by Gd according to Cd_1__−_*_x_*Gd_1+_*_x_*, *x *< 0.02, was observed. The two-phase field β-Gd + CdGd can be considered as a large miscibility gap which cuts off a continuous solid solution (with a second-order phase transition) between these two phases. Samples with major amounts of Cd_1__−_*_x_*Gd_1+_*_x_*, were used to show the correlation of the lattice parameter *a* with the composition. The increasing Gd content goes along with an increase of the lattice parameter which is in perfect agreement with the relative atomic radii of Gd and Cd; the excellent linear fit (Fig. 9) corresponds with Vegard’s rule.

## 4 Conclusions

The complete Cd–Gd phase diagram was reinvestigated by a combination of powder-XRD, SEM and DTA; it is presented in Fig. 1. Based on the present results, the homogeneity ranges of the altogether seven intermetallic compounds were determined and listed in Table 4. Moreover, the extended solid solubility limits of Cd in the low- and high temperature modification of Gd were determined at 4.6 and 22.6 at.%, respectively. The addition of Cd stabilizes the high-temperature modification β-Gd down to 745 °C where it decomposes in terms of a eutectoid reaction.

Moreover, a new compound was found with a stoichiometry of “Cd_8_Gd”, decomposing peritectically at 465 °C. Its corresponding powder-XRD pattern is given in Fig. 3. The peritectic decomposition of Cd_58_Gd_13_ could not be determined as given by Ref. [14] but is assumed that this phase forms through a peritectoidic reaction below 700 °C. It was obtained that the solubility of Cd in Cd_45_Gd_11_ increases rather extensively before the phase decomposes at 808 °C. A phase transformation of Cd_2_Gd was previously indicated by Bruzzone et al. [14] and confirmed according to the present DTA results. However, attempts to quench the high temperature modification β-Cd_2_Gd were not successful although attempts are continuing to clarify its structure. It was observed that the corresponding low-temperature modification α-Cd_2_Gd crystallizes in the simple AlB_2_-type, a fact that was previously discussed for isomorphous α-Cd_2_Pr [10]. A partial substitution of Cd by Gd according to Cd_1__−_*_x_*Gd_1+_*_x_*, *x *< 0.02, was observed in CdGd with CsCl-structure. Actually, it was described that the defect mechanism corresponds to Vegard’s rule (Fig. 9).

## Footnotes

1 National Institute of Standards and Technology, Gaithersburg, MD, USA
